# Deep-Learning for the Diagnosis of Esophageal Cancers and Precursor Lesions in Endoscopic Images: A Model Establishment and Nationwide Multicenter Performance Verification Study

**DOI:** 10.3390/jpm12071052

**Published:** 2022-06-27

**Authors:** Eun Jeong Gong, Chang Seok Bang, Kyoungwon Jung, Su Jin Kim, Jong Wook Kim, Seung In Seo, Uhmyung Lee, You Bin Maeng, Ye Ji Lee, Jae Ick Lee, Gwang Ho Baik, Jae Jun Lee

**Affiliations:** 1 Department of Internal Medicine, Gangneung Asan Hospital, University of Ulsan College of Medicine, Gangneung 25440, Korea; gong-eun@hanmail.net; 2 Department of Internal Medicine, Hallym University College of Medicine, Chuncheon 24253, Korea; doctorssi@kdh.or.kr (S.I.S.); baikgh2@hanmail.net (G.H.B.); 3 Institute for Liver and Digestive Diseases, Hallym University, Chuncheon 24252, Korea; 4 Institute of New Frontier Research, Hallym University College of Medicine, Chuncheon 24253, Korea; iloveu59@hallym.or.kr; 5 Division of Big Data and Artificial Intelligence, Chuncheon Sacred Heart Hospital, Hallym University College of Medicine, Chuncheon 24253, Korea; 6 Department of Internal Medicine, Kosin University College of Medicine, Busan 49267, Korea; forjkw@gmail.com; 7 Department of Internal Medicine, Pusan National University School of Medicine and Biomedical Research Institute, Pusan National University Yangsan Hospital, Yangsan 50615, Korea; pmcac@hanmail.net; 8 Department of Internal Medicine, Inje University Ilsan Paik Hospital, Goyang 10380, Korea; jongman12@gmail.com; 9 Department of Medicine, Hallym University College of Medicine, Chuncheon 24253, Korea; dldjaud@gmail.com (U.L.); ybmaeng@gmail.com (Y.B.M.); 10 Department of Biomedical Science, Hallym University, Chuncheon 24252, Korea; j_wise@naver.com; 11 Department of Life Science, Hallym University, Chuncheon 24252, Korea; woehf96@naver.com; 12 Department of Anesthesiology and Pain Medicine, Hallym University College of Medicine, Chuncheon 24253, Korea

**Keywords:** convolutional neural network, deep learning, endoscopy, esophageal cancers

## Abstract

Background: Suspicion of lesions and prediction of the histology of esophageal cancers or premalignant lesions in endoscopic images are not yet accurate. The local feature selection and optimization functions of the model enabled an accurate analysis of images in deep learning. Objectives: To establish a deep-learning model to diagnose esophageal cancers, precursor lesions, and non-neoplasms using endoscopic images. Additionally, a nationwide prospective multicenter performance verification was conducted to confirm the possibility of real-clinic application. Methods: A total of 5162 white-light endoscopic images were used for the training and internal test of the model classifying esophageal cancers, dysplasias, and non-neoplasms. A no-code deep-learning tool was used for the establishment of the deep-learning model. Prospective multicenter external tests using 836 novel images from five hospitals were conducted. The primary performance metric was the external-test accuracy. An attention map was generated and analyzed to gain the explainability. Results: The established model reached 95.6% (95% confidence interval: 94.2–97.0%) internal-test accuracy (precision: 78.0%, recall: 93.9%, F1 score: 85.2%). Regarding the external tests, the accuracy ranged from 90.0% to 95.8% (overall accuracy: 93.9%). There was no statistical difference in the number of correctly identified the region of interest for the external tests between the expert endoscopist and the established model using attention map analysis (*P* = 0.11). In terms of the dysplasia subgroup, the number of correctly identified regions of interest was higher in the deep-learning model than in the endoscopist group, although statistically insignificant (*P* = 0.48). Conclusions: We established a deep-learning model that accurately classifies esophageal cancers, precursor lesions, and non-neoplasms. This model confirmed the potential for generalizability through multicenter external tests and explainability through the attention map analysis.

## 1. Introduction

Esophageal cancer is the seventh most commonly occurring cancer and the sixth leading cause of cancer-related death worldwide [1]. The management of esophageal cancers has been challenging because most cancers were detected incidentally at locally advanced stages, and even superficial esophageal cancers have the potential for lymph node metastasis due to an abundant lymphatic-capillary plexus in the mucosa or submucosa of the esophagus [2,3]. The standard treatment for esophageal cancer has been radical resection. However, surgery itself carries significant morbidity and mortality, and endoscopic resection is considered an alternative treatment for the subset of superficial esophageal cancers or precursor lesions due to its non-invasiveness, fast recovery, and high post-procedural quality of life [3,4]. Therefore, the early detection and diagnosis of esophageal cancers or precursor lesions are important [5].

However, there has been no standardized screening method for the early diagnosis of esophageal cancers [6]. Incidental detection of esophageal cancers or precursor lesions during upper gastrointestinal endoscopic examination has been the main diagnostic pathway [2]. However, suspicion of the lesion and prediction of the histologic diagnosis of esophageal cancers or premalignant lesions in endoscopic images are not perfect, and early cancers with only subtle mucosal changes pose a diagnostic challenge with white-light imaging alone. Although image-enhanced endoscopy or chromoendoscopy using Lugol’s solution might enhance the diagnostic performance of esophageal cancers, these modalities are not always possible, and inspection with white-light imaging has been the main screening method for routine diagnostic endoscopic procedures [2,7]. Moreover, image-enhanced endoscopy inevitably incurs inter-observer or intra-observer variability and requires substantial time for learning. The esophagus is a physiologically narrow space, and meticulous observation of the mucosa is difficult because of normal peristaltic movements and remnant saliva or mucus. A patient’s heartbeat or breathing also makes it difficult to securely inspect or detect the lesions [2].

**Figure 1:**
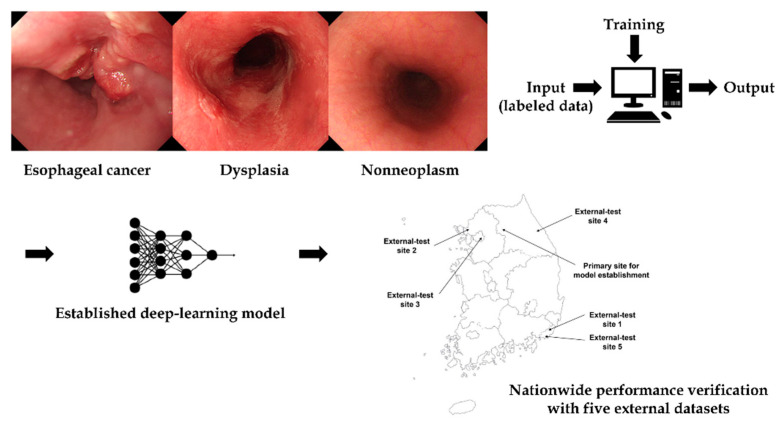
Schematic flow of this study.

The local feature selection and optimization functions of the model enabled an accurate analysis of images in deep learning. The authors previously performed a diagnostic test accuracy meta-analysis for the computer-aided diagnosis of esophageal cancers or neoplasms [2]. The main metric for this study was the pooled sensitivity or specificity. Computer-aided diagnosis models showed high sensitivity or specificity for the diagnosis of esophageal cancers or neoplasms, and the lesion classifying accuracy was also reported in the included studies. However, all the studies classified lesions into only two classes with the adoption of different class standards (i.e., early cancer vs. normal mucosa/Barrett’s neoplasias vs. non-dysplastic Barrett’s esophagus/superficial cancer vs. non-cancer, etc.), which limits the real-clinic application [2]. The purpose of this study was to establish a deep-learning model to classify esophageal cancers, precursor lesions, and non-neoplasms using white-light endoscopic images. Additionally, a nationwide prospective multicenter performance verification was conducted to confirm the possibility of real-clinic application (input data preparation→deep-learning model training and establishment→external performance validation) (Figure 1).

## 2. Methods

### 2.1. Collection and Construction of the Training and Internal-Test Datasets

The authors collected histologically confirmed images from consecutive patients with any type of esophageal cancer or precursor lesions found during upper gastrointestinal endoscopy between 2010 and 2021 in the Chuncheon Sacred Heart hospital to reflect the real-clinic setting. Endoscopic stillcut images were collected from the in-hospital database in JPEG format, with a minimum resolution of 640 × 480 pixels. Only white-light imaging data were collected, and images out of focus or low resolution disabling their proper classification were excluded, as previously described [8,9,10]. All the data collection process was handled by expert endoscopists (E.J.G., C.S.B., and G.H.B.). Finally, a total of 5162 endoscopic images were collected for the training and internal testing. The training and internal-testing datasets were randomly divided into a ratio of 8.5:1.5 (Table 1).

### 2.2. Deep-Learning Tool Used for the Model Establishment

No-code deep-learning tool “Neuro-T” version 2.3.2 (Neurocle Inc., Seoul, South Korea) was used in this study. This can establish deep-learning models for image recognition and classification using a software algorithm that analyses the features of the dataset and self-discovers optimal hyperparameters, thus making it easy for non-experts to build the best performance models [8]. The entire building process of the deep-learning models was approached by simply clicking the menus based on user-friendly graphical user interfaces in on-premise software.

### 2.3. Preprocessing of Collected Training or Internal-Testing Images and Training Parameters

Individual identifiers in the collected images were de-identified before training. The no-code deep-learning tool provides an image resizing transformation function for input images. Users can select multiple modes for the resize transformation of input data, such as “nearest”, “linear”, “cubic”, or “area”. In this study, all images were resized with a resolution of 512 × 480 pixels before training. This no-code deep-learning tool has its own data augmentation functions; however, it does not provide user-selectable options for the classification task.

### 2.4. Training of the Deep-Learning Model

The 5162 endoscopic images were uploaded into the no-code platform tool. Images were then randomly divided into training and internal-test sets at a ratio of 8.5:1.5. After the selection of the data preprocessing options, including “image resize transformation”, as described above, the deep-learning model was trained with specific setting configurations for self-learning. This also offers options for selecting the level of training time based on the available graphic processing units (with four categories: fast, level 1, 2, or 3) and a range of inference speeds based on batch size (three categories: level 1, 2, or 3). Multiple experiments were conducted to identify the best performance deep-learning model based on various hyperparameters in the no-code deep-learning tool. The hardware system used for training the deep-learning models included four RTX 2080 Ti graphics processing units, dual Xeon central processing units, and 256 GB RAM.

### 2.5. Datasets for Nationwide Multicenter Prospective External-Tests

To guarantee the generalizability of performance for the newly established deep-learning model, nationwide multicenter prospective performance verification tests were conducted. These external-test sets, including 836 images, were collected from consecutive patients who underwent upper gastrointestinal endoscopy at the five different University Hospitals (Pusan National University Yangsan Hospital, Inje University Ilsan Paik Hospital, Hallym University Kangdong Sacred Heart hospital, Ulsan University Gangneung Asan hospital, and Kosin University hospital) (Supplementary Figure S1) from 2018 to 2021 (Table 2). All the images were mutually exclusive from those of the training or internal-test dataset.

### 2.6. Primary Performance Metric and Statistics

The primary performance metric was the external-test accuracy. Additional performance metrics were as follows: precision or positive predictive value (defined as (true positive/true positive + false positive)), recall or sensitivity (defined as (true positive/true positive + false negative)), and F1 score (2 × precision × recall/precision + recall). The receiver operating characteristic (ROC) curve was generated and area under the curve (AUC) was also calculated. The number of correctly identified regions of interest for the external-test images between an expert endoscopist and the established model using an attention map was compared through Fisher’s exact test. A *P* < 0.05 (two-tailed) was defined as statistically significant. Fisher’s exact test was performed using SPSS version 24.0. (IBM Corp., Armonk, NY, USA). This study was conducted in accordance with the Declaration of Helsinki and approved by the Institutional Review Board of Chuncheon Sacred Heart Hospital (2021-09-008).

### 2.7. Attention Map for Explainability

A gradient-weighted class activation map (Grad-CAM) was basically implemented into the layer of a neural network to localize the discriminative regions used by the no-code deep-learning tool to determine the specific class in the given images. Grad-CAM was generated for all the external-test images and analyzed to gain the explainability. The ground truth of the lesion in the given images was manually labeled using a square box by expert endoscopists (E.J.G., G.H.B.). The number of correctly identified regions of interest for the external-test images between an expert endoscopist (C.S.B.) and the established model was statistically compared. This was not a detailed segmentation learning analysis; however, it aimed to determine whether or not the established model classified the given lesions based on the correct region of interest in the given images.

## 3. Results

### 3.1. Characteristics of the Datasets

Among the training and internal-test datasets, 17% (878/5162) of the images were esophageal cancers, whereas only 1.3% (66/5162) were dysplastic lesions (Table 1). For the external-tests, 62.2% (520/836) and 5.0% (42/836) of the images were determined to be esophageal cancers and dysplastic lesions, respectively. Various class distributions were observed for each external-test site, with esophageal cancers accounting for 33.3% to 80.4% and dysplasias from 2.4% to 7.3%, respectively (Table 2).

### 3.2. Classification Performance of the Established Deep-Learning Model

**Figure 2:**
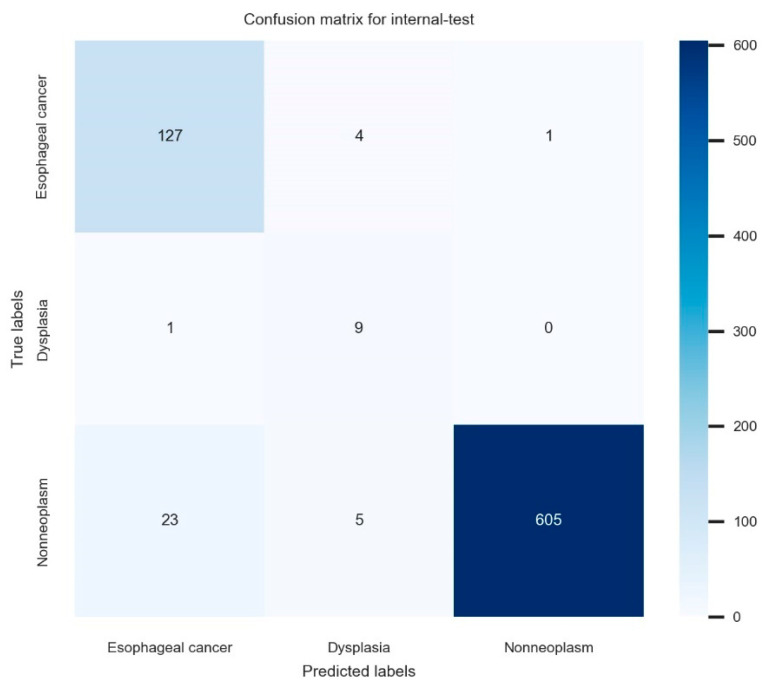
The confusion matrix for the established model in the internal test.

The established model reached 95.6% (95% confidence interval: 94.2–97.0%) internal-test accuracy, 78.0% (75.1–80.9%) average precision, 93.9% (92.2–95.6%) average recall, and 85.2% (82.7–87.7%) F1 score in the internal-test. The total training time was 629 min. The confusion matrix for the established model in the internal test is illustrated in Figure 2. The ROC curve with per-class AUCs for the internal test is illustrated in Supplementary Figure S2 (AUC for esophageal cancer: 0.95 (range: 0.90–0.99), dysplasia: 0.80 (0.63–0.97), and non-neoplasm: 0.96 (0.93–0.99)). The detailed information of the hyperparameters in the established model is as follows; batch size: 40, epochs: 62, number of layers: 18, optimizer: momentum, input height, and width: 480 × 512, all images were resized with inter-linear interpolation, initial learning rate: 0.00146. The detailed performance metrics of the internal test are shown in Table 3.

### 3.3. Nationwide Prospective Multicenter Performance Verification

**Figure 3:**
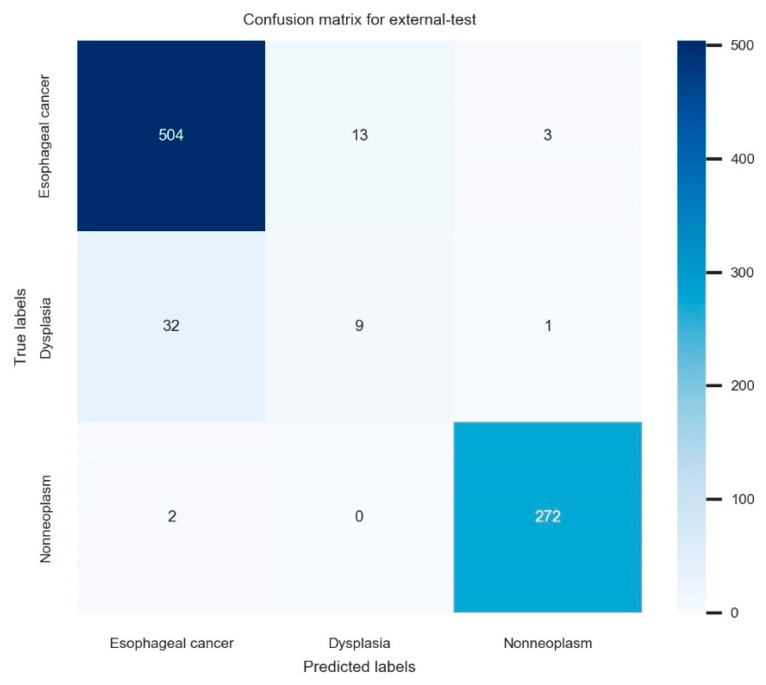
The confusion matrix for the established model in the external test.

For the 836 images in the nationwide prospective multicenter performance external-test, the established model showed 93.9% (95% confidence interval: 92.3–95.5%) accuracy, 77.7% (74.9–80.5%) average precision, 72.5% (69.5–75.5%) average recall, and 75.0% (72.1–77.9%) F1 score. The primary performance metric in this study, which is external accuracy, showed robust values irrespective of the various class distributions in each external-test site, ranging from 90.0% to 95.8%. The confusion matrix for the established model in the external test is illustrated in Figure 3. The ROC curve with per-class AUCs for the external-test is illustrated in Supplementary Figure S3 (AUC for esophageal cancer: 0.97 (range: 0.94–0.99), dysplasia: 0.71 (0.58–0.84), non-neoplasm: 0.97 (0.95–0.99)). The detailed performance metrics of the external test are shown in Table 3.

### 3.4. Attention Map for the Explainability

**Figure 4:**
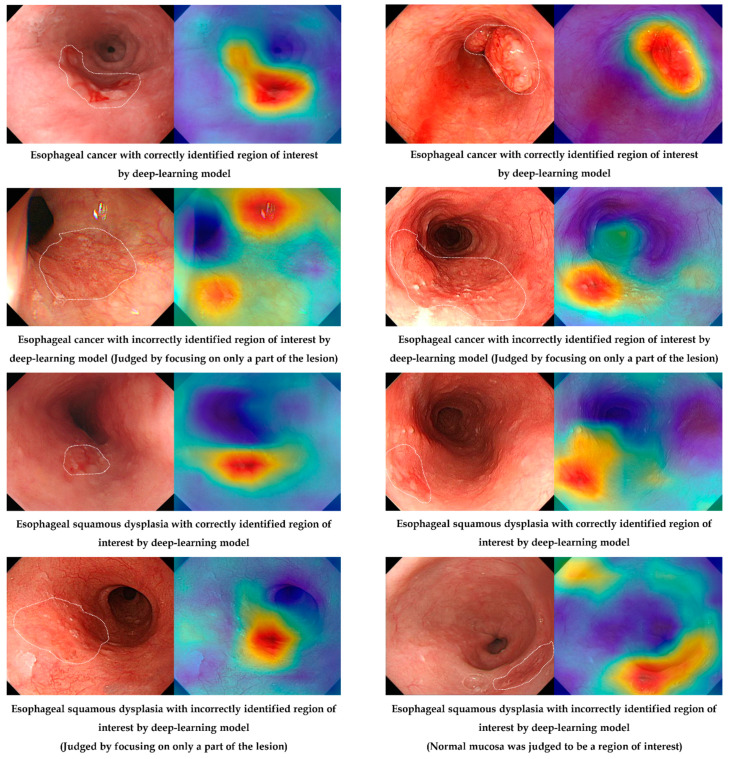
Correctly or incorrectly determined samples in the external test by the established deep-learning model.

Figure 4 shows the correctly and incorrectly determined samples in the external test by the established deep-learning model. Although an incorrectly identified region of interest exists in the deep-learning model (judged by focusing on only a part of the lesion), the characteristic area of the lesion was noted by an established model in most cases.

The number of correctly identified regions of interest for the external-test images between the expert endoscopist and the established model was statistically compared, and there was no statistical difference in the number of correctly identified regions of interest for the external-test images between the expert endoscopist and the established model using Grad-CAM analysis (*P* = 0.11). In terms of the dysplasia subgroup, the number of correctly identified regions of interest was higher for the deep-learning model than for endoscopists; however, the difference was not statistically significant (92.9% vs. 85.7%, *P* = 0.48) (Supplementary Table S1).

## 4. Discussion

The established deep-learning model in this study accurately classifies esophageal cancers, precursor lesions, and non-neoplasms in endoscopic images with an overall accuracy of 93.9% (the accuracy ranged from 90.0% to 95.8%) in the external verification tests. This model confirmed the potential for generalizability through the multicenter external tests and explainability through the attention map analysis. However, some cases for incorrectly determined regions of interest in the given images by the deep-learning model were detected, and there was no statistically significant difference in the proportion of correctly identified regions of interest between expert endoscopists and the established model. To the best of our knowledge, this model is the first deep-learning model that considers all lesions at the developmental stage of esophageal cancers (non-neoplasms, precursor lesions, and esophageal cancers).

Previous studies classified given lesions into only two classes by adopting different class standards, such as early cancer vs. normal mucosa/Barrett’s neoplasias vs. non-dysplastic Barrett’s esophagus/superficial cancer vs. non-cancer, etc., which limit the real practice application [2]. In East Asia, the majority of esophageal cancers are histologically squamous cell carcinomas. Considering that esophageal squamous dysplasia is the only histopathology that predicts the development of esophageal squamous cell carcinoma, model establishment distinguishing three classes is clinically useful and reasonable [11].

In the context of the amount of training data, the amount of data needed to reasonably approximate the unknown underlying mapping function in deep learning is unknown [12,13]. In general, too little training data would result in a poor approximation. In terms of the complexity of the model, an over-constrained model would underfit the small training dataset, whereas an under-constrained model would overfit the training data; however, both can lead to poor performance [13,14]. A large amount of data is not necessarily good for training. A data-volume-dependent performance plateau occurs, which is related to whether the data have sufficient features and the complexity of the background model [15]. Regardless of the relationship between the volume of data and the complexity of the model, the quality and class distribution of the data is also important, and it is certain that an established model based on training data that cannot reflect a real-clinic setting would mislead the users [12,13,16]. Therefore, the authors collected the histologically confirmed images from consecutive patients with any type of esophageal cancer or precursor lesions found during upper gastrointestinal endoscopy to reflect a real situation. Various prevalence values of squamous dysplasia ranging from 3% to 38% have been reported in the literature [17,18].^,^ However, a small distribution (1.3%) of esophageal dysplasia was noted in the training dataset in our study. Considering the decreasing trend of esophageal squamous cell carcinomas in Korea, we think this value might reasonably reflect the current situation of endoscopic screening for esophageal cancers or precursor lesions in Korea [11,19].

Making an artificial intelligence model (modeling) is similar to problem-solving and making statistical functions designed to predict the outcomes of certain problems. After producing a hypothetical statistical model, the method of confirmation of the clinical usefulness is external validation. However, no single study has performed external validation in the relevant studies according to the previous meta-analysis [2]. Only recently published studies presented the external-test performance; however, this model showed slightly lower accuracy compared to ours and premalignant lesions were not considered [20]. Too little external-test data would result in an optimistic and high variance estimation of model performance. However, we enrolled 836 images with various features from five different institutions in our study and showed consistently high performances irrespective of the external-test sites.

Another interesting point in this study would be that a no-code deep-learning tool was used in establishing the model. The traditional method of creating an artificial intelligence model is to connect to a computing platform and create a program file through coding using a programming language. However, clinicians often lack this professional expertise to directly connect clinical data to the model establishment [8]. The no-code deep-learning tool makes it possible for clinicians to create high-quality deep-learning models. These tools are easy to use and help in producing deep-learning models in efficient ways. After self-learning and finding optimal parameters and neural architectures, this tool provides models ready for direct deployment in real clinics [8]. Considerably less time was needed for model establishment compared to the traditional method, and only 629 min were required for the total training in this study.

The current study established a high-performance deep-learning model with confirmation of the potential for generalizability and explainability. However, several inevitable limitations were detected. First, the majority of the training images included squamous cell carcinoma, except for only two adenocarcinomas, which is limited to the screening of Barrett’s neoplasias. Considering that squamous cell carcinoma is still the major histologic type of esophageal cancer in East Asia, the clinical utility of this model is expected to be valid. Second, the diagnostic performance of the established model for squamous dysplasia was relatively lower than that of other classes. Figure 3 shows that misclassified dysplasias were diagnosed as cancer by the deep-learning model. This means misclassified dysplasia were not determined by nonneoplasm and clinical upstaging by the deep-learning model. This is a role of alarming the clinician about the possibility of cancer for dysplasias, which is a role of upstaging rather than clinical down-staging. Given that only a small amount of data were included in the training for dysplasias, further enrollment of this subclass and changing the distribution of all the included images would enhance the per-class performances. In the future, it may be possible to improve the model’s performance by obtaining a dataset with a higher proportion of adenocarcinoma and dysplasia in order to generalize the model’s performance.

## 5. Conclusions

In conclusion, an established deep-learning model accurately classifies esophageal cancers, precursor lesions, and non-neoplasms. This model confirmed the potential for generalizability through multicenter external tests and explainability through the attention map analysis.

## Figures and Tables

**Table 1:** Distribution of the collected images according to the training and internal-test datasets.

	Training Dataset (Number of Images)	Internal-Test Dataset (Number of Images)	Total (Number of Images)
Overall	4387	775	5162
Esophageal cancer	746	132	878 (17.0%)
Dysplasia	56	10	66 (1.3%)
Nonneoplasm	3585	633	4218 (81.7%)

**Table 2:** Distribution of the collected images for the external-test datasets.

Number of Images (%)	Overall	External-Test Dataset 1 (Pusan National University Yangsan Hospital)	External-Test Dataset 2 (Inje University Ilsan Paik Hospital)	External-Test Dataset 3 (Hallym University Kangdong Sacred Heart Hospital)	External-Test Dataset 4 (Ulsan University Gangneung Asan Hospital)	External-Test Dataset 5 (Kosin University Hospital)
Overall	836	119	126	78	363	150
Esophageal cancer	520 (62.2%)	48 (40.3%)	69 (54.8%)	26 (33.3%)	292 (80.4%)	85 (56.7%)
Dysplasia	42 (5.0%)	8 (6.7%)	3 (2.4%)	3 (3.8%)	17 (4.7%)	11 (7.3%)
Nonneoplasm	274 (32.8%)	63 (52.9%)	54 (42.9%)	49 (62.8%)	54 (14.9%)	54 (36.0%)

**Table 3:** Summary of the internal- and external-test performance metrics for the established deep-learning model.

(Values with 95% Confidence Interval)	Accuracy (%)	Precision (%)	Recall (%)	F1 Score (%)
Internal-test performance (*n* = 775)	95.6 (94.2–97.0)	78.0 (75.1–80.9)	93.9 (92.2–95.6)	85.2 (82.7–87.7)
Overall external-test performance (*n* = 836)	93.9 (92.3–95.5)	77.7 (74.9–80.5)	72.5 (69.5–75.5)	75.0 (72.1–77.9)
External-test performance 1 (*n* = 119)	95.8 (92.2–99.4)	90.7 (85.5–95.9)	82.6 (75.8–89.4)	86.5 (80.4–92.6)
External-test performance 2 (*n* = 126)	95.2 (91.5–98.9)	64.0 (55.6–72.4)	65.2 (56.9–73.5)	64.6 (56.3–72.9)
External-test performance 3 (*n* = 78)	94.9 (90.0–99.8)	94.3 (89.2–99.4)	65.4 (54.8–76.0)	77.2 (67.9–86.5)
External-test performance 4 *(n* = 363)	94.8 (92.5–97.1)	78.6 (74.4–82.8)	73.8 (69.3–78.3)	76.1 (71.7–80.5)
External-test performance 5 (*n* = 150)	90.0 (85.2–94.8)	68.5 (61.1–75.9)	67.7 (60.2–75.2)	68.1 (60.6–75.6)

## Data Availability

All the data are accessible and available upon request by the corresponding author. Access to data: all investigators have access to the final dataset.

## Supplementary Materials

The following supporting information can be downloaded at: https://www.mdpi.com/article/10.3390/jpm12071052/s1, Figure S1: The geographic location of the primary site for the model establishment and multicenter sites for the external-tests; Figure S2: The receiver operating characteristic curve with per-class area under the curve for the internal-test. AUC, area under the curve. The maximal values are described in the right panel; Figure S3: The receiver operating characteristic curve with per-class area under the curve for the external-test. The maximal values are described in the right panel. AUC, area under the curve. The maximal values are described in the right panel; Table S1: The number of correctly identified regions of interest for the external-test images between expert endoscopist and the established model.

## Abbreviations

ROC	receiver operating characteristic
AUC	area under the curve
Grad-CAM	gradient-weighted class activation map
