# Supplementary material for: Deep-Learning for the Diagnosis of Esophageal Cancers and Precursor Lesions in Endoscopic Images: A Model Establishment and Nationwide Multicenter Performance Verification Study
Source: J Pers Med. 2022 Jun 27;12(7):1052. doi: 10.3390/jpm12071052 (PMC9320232; doi:10.3390/jpm12071052)
Supplement: Supplementary file 1 [file jpm-12-01052-s001.zip › jpm-1752156-supplementary.pdf]

Supplementary materials:

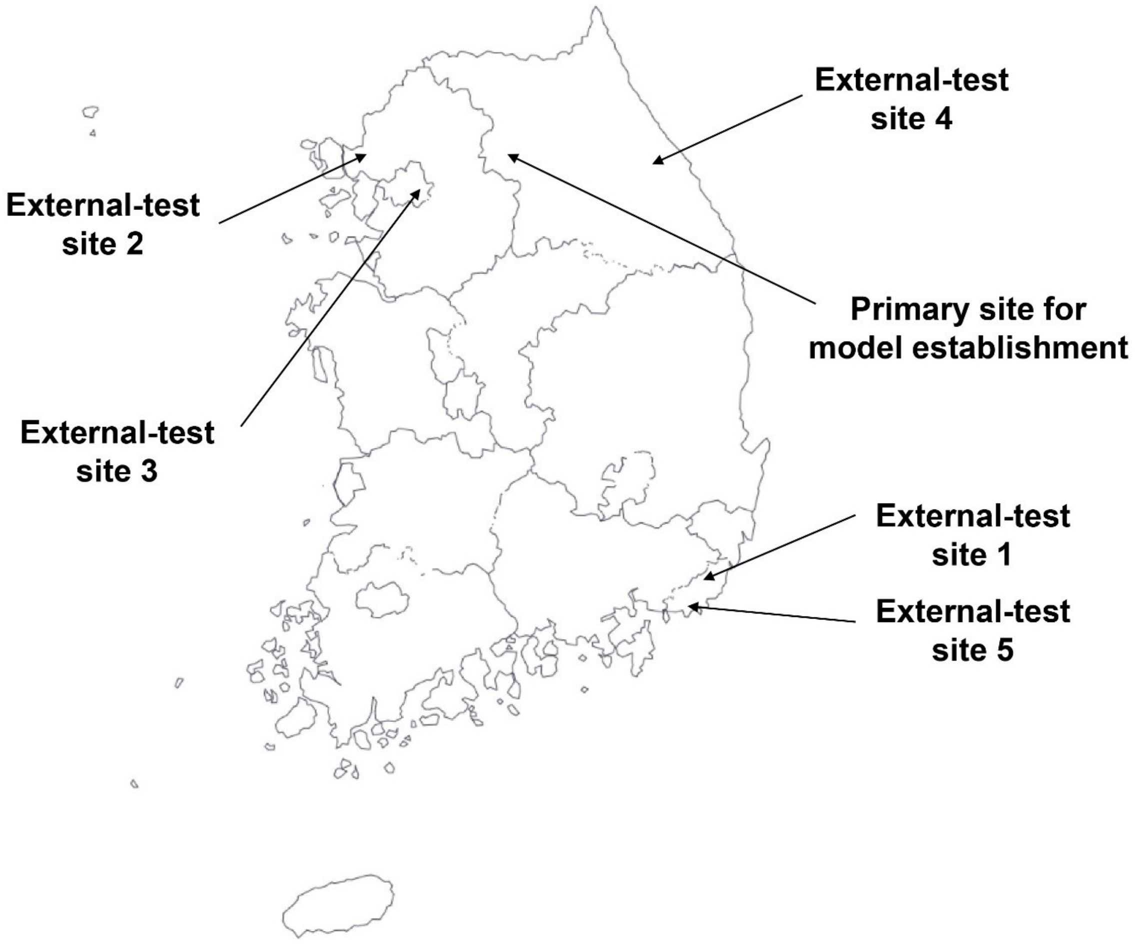

Figure S1. The geographic location of the primary site for the model establishment and multicenter sites for the external-tests.

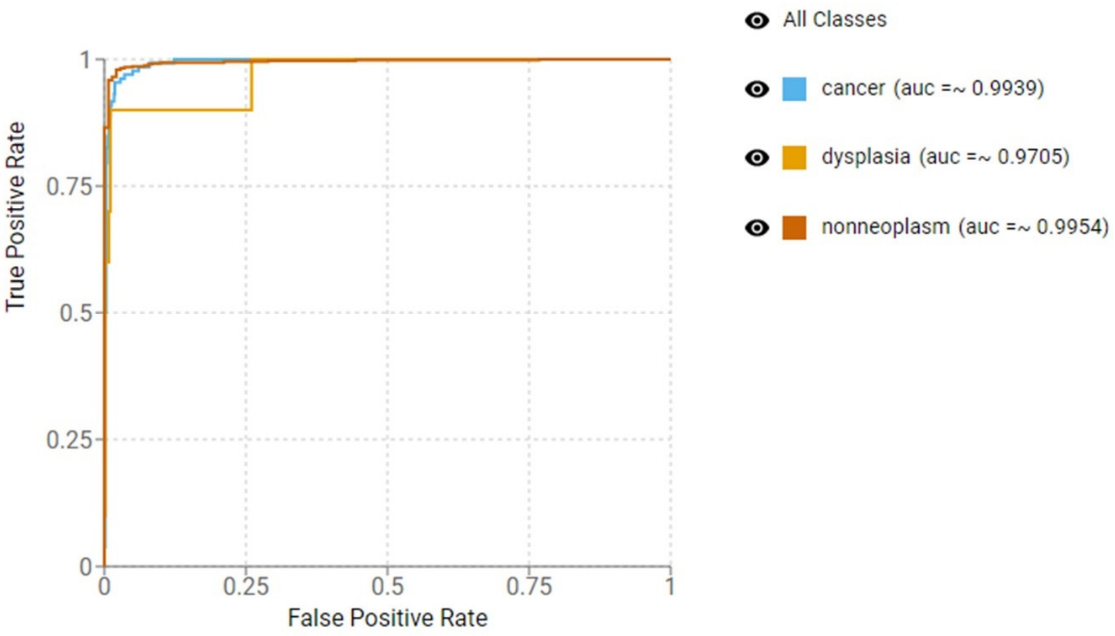

Figure S2. The receiver operating characteristic curve with per-class area under the curve for the internal-test. AUC, area under the curve. The maximal values are described in the right panel.

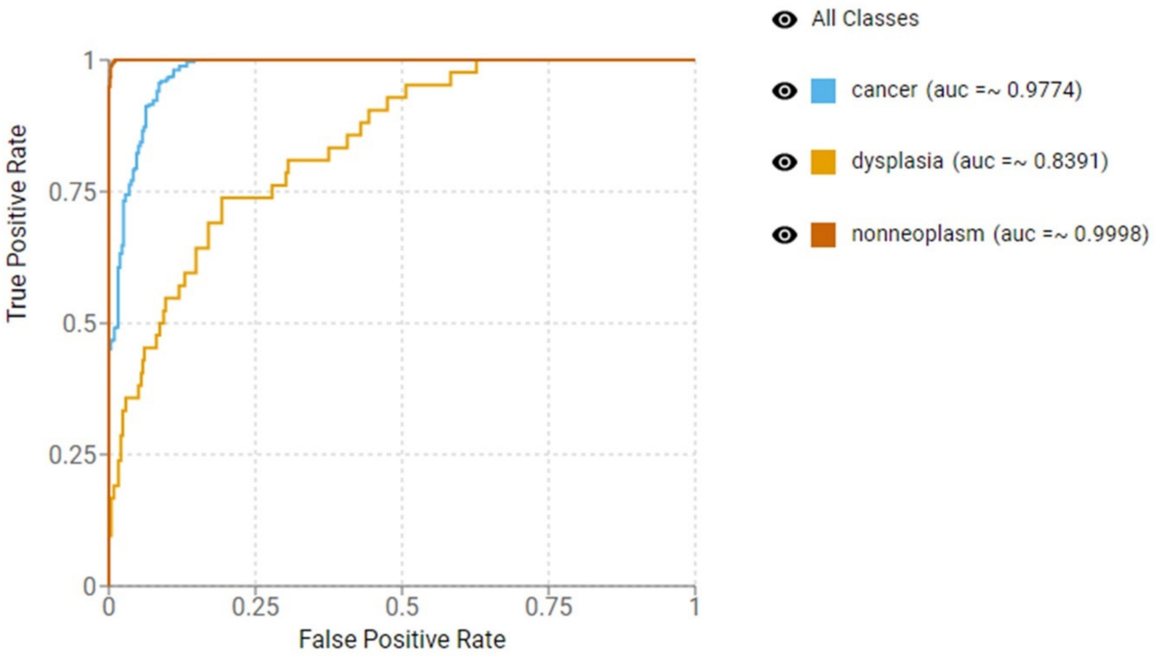

Figure S3. The receiver operating characteristic curve with per-class area under the curve for the external-test. The maximal values are described in the right panel. AUC, area under the curve. The maximal values are described in the right panel.

Table S1. The number of correctly identified regions of interest for the external-test images between expert endoscopist and the established model.

	Overall (number of images)	Established deep-learning model (correctly identified number, %)	Expert endoscopist (correctly identified number, %)
Overall	836	826 (98.8%)	819 (98.0%)
Esophageal cancer	520	513 (98.7%)	509 (97.9%)
Dysplasia	42	39 (92.9%)	36 (85.7%)
Nonneoplasm	274	274 (100%)	274 (100%)
